# Editorial

**DOI:** 10.22336/rjo.2017.28

**Published:** 2017

**Authors:** Zemba Mihail

**Affiliations:** * Editor-In-Chief

The Fourth Congress of the Romanian Society of Cataract and Refractive Surgery, connected with the Annual Conference of the Romanian Retina Society took place in the period 22-25 of June 2017, in Eforie Nord.

The association of these two conferences, already a tradition, was again a great success, with an audience of more than five hundred ophthalmologists from Romania and from abroad.

The event started on Thursday with an interesting symposium about the role of aflibercept in retinal pathology. Horia Stanca, MD, PhD, and Prof. Mihnea Munteanu, MD, PhD, shared their experience with this type of treatment.

The Conference continued with three very interesting lectures: Assoc. Prof. Florian Balta talked about vitamin D and retina, Daniel Branisteanu, MD, PhD, presented an update about the surgical treatment in retinology and Horia Stanca, MD, PhD, approached a very difficult issue: education in ophthalmology, talking about his experience with the educational system for residents in the USA.

The following day, the Congress of Romanian Society of Cataract and Refractive Surgery began. There were two days with a very dense schedule, very interesting topics being approached during the sessions: Ngenuity 3D Visualization System Platform, both for anterior and posterior pole surgery, new types of premium intraocular lenses, the role of vitreoretinal surgeon in managing ocular trauma, premium personalized intraocular lens and others.

The session about nightmares in ophthalmology was very much appreciated, during which, experienced surgeons shared their experience with very complicated cases.

The international participation was important, with interesting presentations of Prof. Usha Chakravarthy - Belfast – on OCT, Juergen Beierl on Refractive Corneal Topography and Prof. Sorcha Ni Dhunhgheill – Antwerp - on cataract surgery in children, bag in the lens technique.

An important event that also took place during this Congress was the election for the Board of Romanian Society of Cataract and Refractive Surgery. Prof. Calin Tataru, MD, PhD, the leader of this Society the last four years, presented a scientific and financial audit. The overall conclusion was that this was a remarkable period, with many achievements: four Congresses of the Society, the joint with the Conference of Romanian Retina Society, the print of the Guide for Cataract Surgery and many others.

The delegates appreciated very much the activity of the former President, so, after the voting, Prof. Calin Tataru, MD, PhD, remained President of Romanian Society of Cataract and Refractive Surgery for the next four years. He suggested and the delegates approved that the former Secretary and Treasurer would remain in duty. Musat Ovidiu, MD, PhD, and Madalina Serban, MD, PhD, were confirmed as Secretary and Treasurer by the delegates.

Finally, a vote for two vice presidents established that Dorin Nicula, MD, PhD, and Mihail Zemba, MD, PhD, would join the Board of Romanian Society of Cataract and Refractive Surgery for the next four years. 

The Editorial Board of Romanian Journal of Ophthalmology wishes great success to all of them.

**Editor-in-Chief** **Mihail Zemba, MD, PhD**

